# Research progress of biological processing of traditional Chinese medicine:fermentation and sprouting

**DOI:** 10.1186/s13020-026-01393-1

**Published:** 2026-05-22

**Authors:** Jia-qi Shi, Xiao-ting Wang, Jia-li Tian, Yu Shi, Ya-zhu Wang, Shu-qing Tian, Fan Zhang, Hui Gao

**Affiliations:** 1 School of Pharmacy, Liaoning University of Traditional Chinese Medicine, Liaoning Dalian, 116600 China; 2 Traditional Chinese Medicine Processing Technology Inheritance Base (Liaoning) of the National Administration of Traditional Chinese Medicine, Dalian, 116600 China; 3 Liaoning Provincial Traditional Chinese Medicine Processing Technology Innovation Center, Dalian, 116600 China

**Keywords:** Biological processing, Traditional Chinese medicine, Fermentation, Sprouting, Processing

## Abstract

**Aim:**

This paper presents a comprehensive review focusing on biological processing of traditional Chinese medicine. It reviews emerging modern fermentation technologies, as well as the two major aspects of biological processing of traditional Chinese medicine, fermentation processing and sprouting processing, covering key areas including their processing techniques, changes in TCM active ingredients, alterations in pharmacodynamic effects, and quality evaluation methods. The purpose of this review is to summarize the progress of TCM bioprocessing technologies, identify shortcomings in existing research, clarify the internal connections between different research areas, and ultimately provide comprehensive and reliable references for the further development of TCM bioprocessing research and its clinical application.

**Materials and methods:**

PubMed, CNKI, Wanfang and CBM were systematically searched using keywords including “fermentation”, “sprouting”, “*Arisaema cum Bile*”, and “*Massa Medicata Fermentata*”. Relevant articles on TCM biological processing were screened and reviewed.

**Results:**

Biological processing contributes significantly to optimizing TCM quality, potentiating efficacy and attenuating toxicity. Nevertheless, its industrialization is limited by unstable microbial strains and incomplete quality assessment systems, while systematic modern scientific elucidations are still lacking for its mechanisms and standardized application.

**Conclusion:**

Biological processing of traditional Chinese medicine has fully exerted the clinical value of TCM and provided an important guarantee for the application of TCM. Future research should clarify its scientific connotation using modern indicators, improve its theoretical system, promote scaled and standardized development, and thereby advance the modernization and internationalization of TCM.

## Introduction

The processing of Chinese herbal medicines is a unique pharmaceutical technology that processes crude drugs according to the TCM theory and individual crude drugs’ nature, and the requirements of drug dispensing, pharmaceutical preparation, and clinical use, has the effects of reducing toxicity and enhancing efficacy, including methods such as stir-baking, carbonizing, steaming, boiling, fermenting, sprouting, and so on. Biological processing of TCM is a new concept summarized by senior specialists in the field of processing on the basis of modern research on traditional preparation methods such as fermenting and sprouting [51]. It is the processing technology that make use of microorganisms or enzymes to change the original physical and chemical property, promote biological efficacy, reduce toxicity of the traditional Chinese medicine decoction pieces and produce new products. It not only includes the traditional natural fermentation, sprouting and other processing technology, but also includes modern methods for preparing new TCM decoction pieces and raw medicinal materials by fermentation and sprouting engineering in modern processing technology [50].

Biological processing of TCM is a new technology of TCM pharmaceuticals formed by inheriting the TCM processing and combining with modern bioengineering. *Semen oryzae cum Monasco*, a traditional fermented Chinese medicine with lovastatin as its core lipid-lowering component, is a typical representative of traditional fermented Chinese medicines. In ancient China, *Semen oryzae cum Monasco* was produced from rice under specific conditions through natural fermentation [155]. Although the role of *Monascus* in the natural environment was not discovered due to the lack of microbiological knowledge at that time, long-term practice has confirmed its effects of strengthening the spleen, aiding digestion, promoting blood circulation, and resolving stasis [148]. With the advancement of modern research, statins such as *monacolin K* produced during the fermentation of *Semen oryzae cum Monasco* have been identified as the material basis for lipid-lowering and blood circulation improvement [15]. Based on this discovery, the traditional fermentation process of *Semen oryzae cum Monasco* has been reformed to ferment with specific strains of *Monascus*. The fermented *Sojae Semen Praeparatum* also exemplifies the integration of traditional fermentation and modern technology. Traditional processing primarily uses black beans as raw materials, utilizing microbial fermentation in natural environments [127, 128, 132, 137]. Modern research has further elucidated the transformation pathways of substances like soy isoflavones during fermentation, confirming that the nutritional value of fermented *Sojae Semen Praeparatum* is significantly enhanced, and glycosides can be maximally converted into aglycones [170]. Additionally, studies have revealed that various microorganisms, including *Bacillus circulans, Aspergillus niger*, and *Rhizopus arrhizus*, play synergistic roles in fermentation. The adoption of pure strain or multi-strain co-fermentation technology can more precisely ensure the flavor, efficacy, and quality stability of *Sojae Semen Praeparatum* [114].

**Fig. 1:**
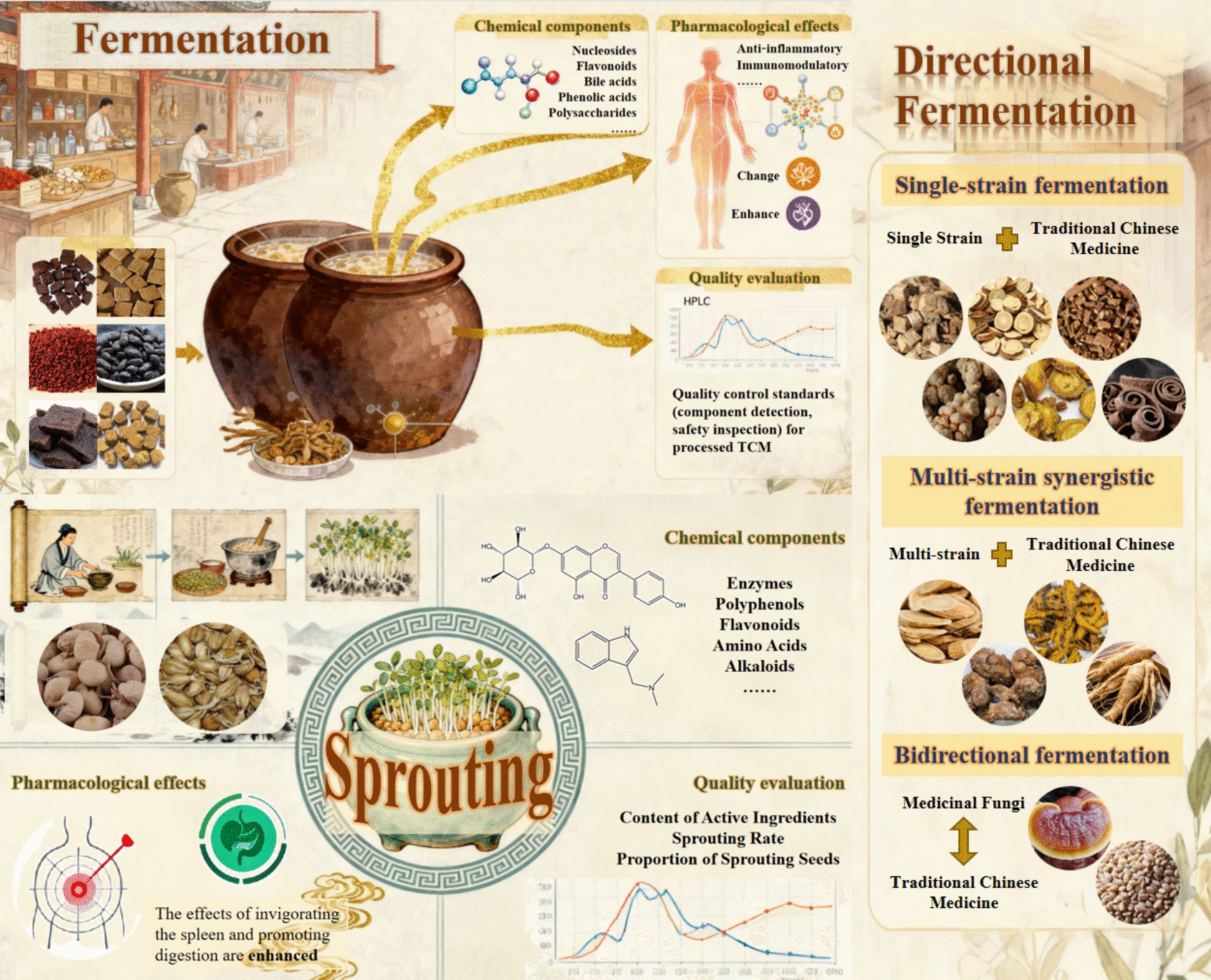
Overview of biological preparation of Chinese medicine

Under the impetus of modernization in TCM, the field of biological processing of TCM has experienced rapid development. Various traditional fermented and sprouted TCM has been studied specifically, such as the microbial community structure and fermentation conditions of *Massa Medicata Fermentata* [181], the bile fermentation detoxification and efficacy enhancement principles of *Arisaema cum Bile* [109], and the active ingredient transformation pathways of *Hordei Fructus Germinatus* [160] and *Sojae Semen Germinatum* [125, 136]. Although existing studies involve process, composition, and pharmacological analysis of certain varieties, there is a lack of systematic integration and review of biological processing technologies. Based on these insights, this article focuses on two core directions, fermentation processing and sprouting processing of TCM, to systematically summarize their processing techniques, composition changes, pharmacological characteristics, and quality control methods. (Fig. 1) The purpose is to review research progress, identify shortcomings, and provide comprehensive and reliable references for the further development and clinical application of biological processing in TCM.

## Preparation technology of Chinese medicine fermentation

### History of preparation technology of Chinese medicine fermentation

**Fig. 2:**
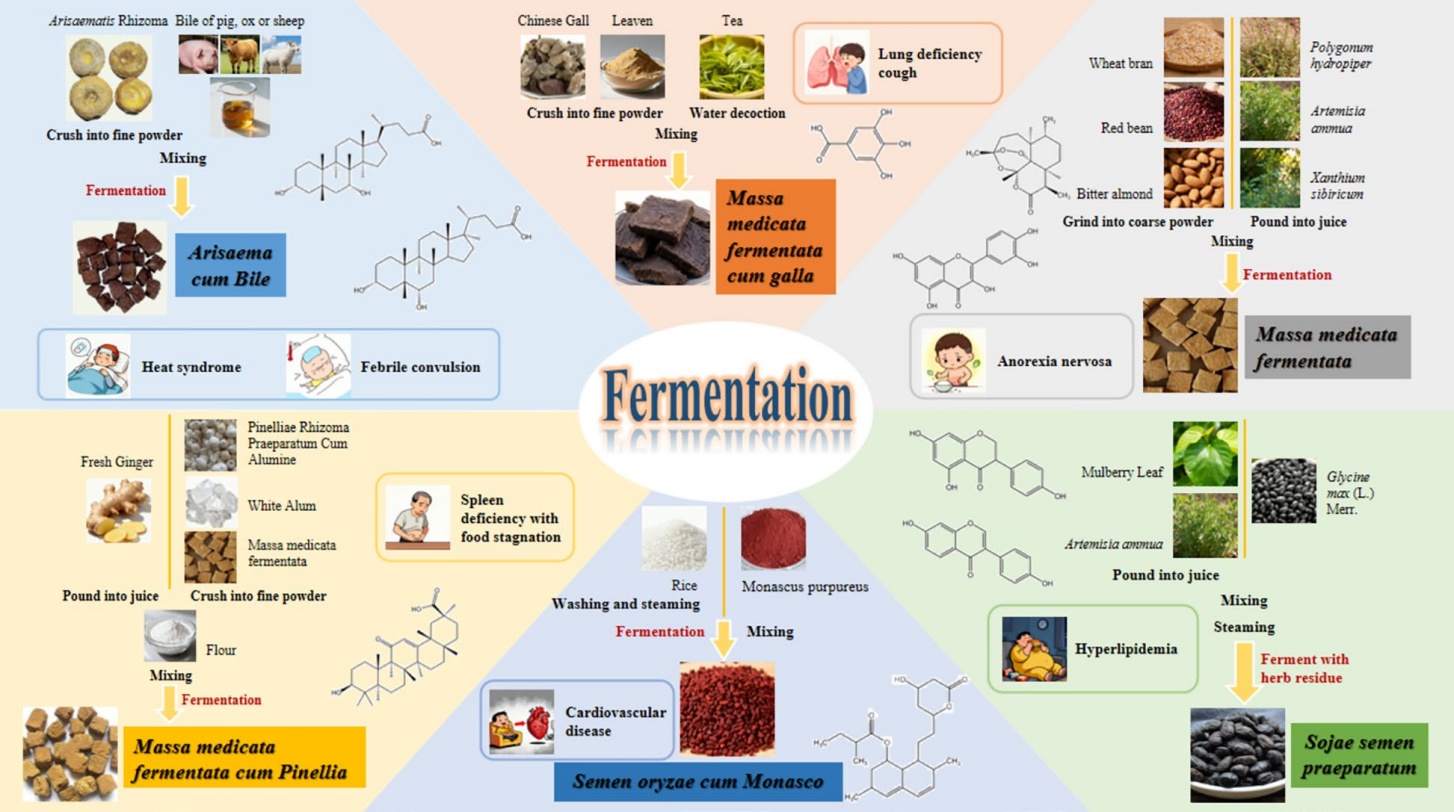
Representative varieties of traditional fermentation of Chinese medicine

Early fermentation refers to the natural process that occurs when fruits or grains are stored. After people first observed it, they began to utilize fermentation technology for brewing wine, which was later successively used to produce foods such as soy sauce, vinegar, and fermented black beans [58, 63]. With the development of fermentation methods, people started using koji as medicine, adding fermented koji to purified traditional Chinese medicines and fermenting them under specific conditions. By leveraging the transformative effects of microorganisms on traditional Chinese medicines, they ultimately produced specialized fermented koji for medicinal use. The earliest recorded use of fermented traditional Chinese medicine pieces for medicinal purposes can be traced back to the Eastern Han Dynasty, when *Treatise on Cold Damage Disorders and Miscellaneous Illnesses* mentioned the use of koji and fragranted soybeans in treating diseases [169, 175]. By the Tang Dynasty, fermented traditional Chinese medicines achieved institutional breakthroughs. China's first officially compiled pharmacopoeia, the *Newly Revised Materia Medica*, incorporated fermentation methods into formal processing standards and explicitly defined their medicinal properties. This initiative not only elevated the academic status of fermented traditional Chinese medicines but also laid the institutional foundation for technological inheritance and variety innovation in later generations [52]. From the Song Dynasty to the Jin and Yuan Dynasties,, the development of traditional fermentation technology reached a new height. The types of koji and their production methods expanded. Additionally, new fermented products that were directly fermented using traditional Chinese medicines emerged, such as *Arisaema cum Bile* and *Galla chinensis Praeparata*, or varieties using fermentation to preserve the medicinal effects, like *Fermented Aconite*. The Ming and Qing dynasties' works such as *Compendium of Materia Medica* and *Great Methods of Processing* recorded the formulations and fermentation methods of various koji, including *Massa Medicata Fermentata*, *Massa Medicata Fermentata cum Pinellia*, and *Massa Medicata Fermentata cum Aquilaria* were recorded. Some of these varieties have been passed down to the present day and are documented in the Pharmacopoeia and local processing specifications [111]. Among them, *Arisaema cum Bile*, *Massa Medicata Fermentata*, *Sojae Semen Praeparatum*, *Massa Medicata Fermentata cum Galla*, *Semen oryzae cum Monasco*, etc., are even commonly used decoction pieces in clinical practice (Table 1) [52]. The emergence of qu preparations greatly enriched the technical system of traditional Chinese medicine processing, expanded the scope of clinical applications, and further enhanced the efficacy and safety of traditional Chinese medicine through processing effects such as enhancing efficacy, reducing toxicity, and altering drug properties, providing important support for traditional pharmaceutical theory and clinical compatibility. This review focuses on these traditional fermented Chinese medicines, with the aim of providing valuable references for relevant research and clinical applications (Fig. 2, Table 2).

**Table 1:** Fermentation varieties recorded in successive dynasties

Variety	Latin name	Dynasty	Literature	Content
Dan Nan Xing	*Arisaema cum Bile*	Song	*General Record of Holy Relief*	The method of “ox bile fermentation” is recorded
		Song	*Key to the Therapeutics of Children's Diseases*	The method of “Prepared in winter, stored in an ox gallbladder, and dried in the shade for a hundred days” of *Arisaema cum Bile* is recorded
		Song	*Effective Prescriptions for Universal Relief*	The first record of “goat bile fermentation” is recorded
		Qing	*Complete Work on Children's Diseases*	The detailed fermentation method of *Arisaema cum Bile* is recorded
Liu Shen Qu	*Massa Medicata Fermentata*	Tang	*Important Methods to Condion the People*	Three kinds of methods for fermenting *Massa Medicata Fermentata* are recorded, mainly for the purpose of brewing wine
		Tang	*Treatise on the Properties of Drugs*	It is the first time that *Massa Medicata Fermentata* has been recorded as a drug name
		Ming	*Enlightening Primer or Materia Medica*	The composition and fermentation method of *Massa Medicata Fermentata* are included
Dan Dou Chi	*Sojae Semen Praeparatum*	Ming	*Compendium of Materia Medica*	The methods for the preparation of *Sojae Semen Praeparatum* by fermentation of *mori* Folium and *artemisiae* Annuae Herba are recorded
		Ming	*Medical Complete Book, Ancient and Modern*	Three different preparation methods were recorded, one of which did not use Chinese herbal adjuvants, and the other two used warm adjuvants Perillae Folium and cold adjuvants Mori Folium respectively
Hong Qu	*Semen oryzae cum Monasco*	Song	*Extensive Records of the Forest of Affairs*	The earliest known method for preparing *Semen oryzae cum Monasco*
		Yuan	*Principles of Correct Diet*	*Semen oryzae cum Monasco* was first recorded in medicine
		Ming	*Compendium of Materia Medica*	The preparation method of *Semen oryzae cum Monasco* is described in detail
Bai Yao Jian	*Massa Medicata Fermentata cum Galla*	Song	*Prescriptions Collected by the Public Pharmacy*	It is recorded that *Massa Medicata Fermentata cum Galla* can be used to treat chronic cough with much phlegm, sore throat, bloody stool, mouth ulcers and other symptoms
		Ming	*Enlightening Primer or Materia Medica*	It is the first time that *Massa Medicata Fermentata cum Galla* is made from *Galla chinensis*
		Ming	*Yixue Rumen*	For the first time, dark plum and alum were added to the formula, with distiller's yeast used as the source of fermentation strains
		Qing	*Huizhitang Jingyan Fang*	It was first proposed to crush *Galla chinensis* and ferment it with white wine
Ban Xia Qu	*Massa Medicata Fermentata cum Pinellia*	Song	*Prescriptions Collected by the Public Pharmacy*	It is recorded that the preparation method of *Massa Medicata Fermentata cum Pinellia* is: “Wash the Pinelliae Rhizoma, grind it into powder, mix it with ginger juice, and make it into leaven.”
		Song	*Key to the Therapeutics of Children's Diseases*	It is recorded that “Pinelliae Rhizoma and ginger are pounded together to make leaven.”
		Ming	*Hanshi Yitong*	It is proposed that *Massa Medicata Fermentata cum Pinellia* is no longer made from Pinelliae Rhizoma powder and ginger, but is prepared by adjusting the compatible drugs according to the needs of diseases

**Table 2:** Preparation technology of Chinese medicine fermentation

Variety	Material	Bioactive compound	Health benefit effect	Underlying molecular mechanism
*Arisaema cum Bile*	*Arisaematis Rhizoma,* Bile of pig, ox or sheep	Bile acids (HCA、CDCA、HDCA…), polysaccharides, flavonoids, alkaloids	The effects of clearing heat and resolving phlegm, as well as calming wind and relieving convulsions	Block oxidative stress and reduce the release of inflammatory factors
*Massa Medicata Fermentata*	Wheat bran, *Polygonum hydropiper*, Red bean, Bitter almond, *Artemisia ammua*, *Xanthium sibiricum*	Quercetin, luteolin, leucocyanidin, arteannuin B	Strengthening the spleen and harmonizing the stomach, promoting digestion and regulating the middle energizer	Promote gastrointestinal motility, regulate intestinal microbiota, and exert anti-inflammatory effects
*Sojae Semen Praeparatum*	Mulberry Leaf, *Artemisia ammua*, *Glycine max (L.) Merr*	Saponin aglycone, delphinidin	Relieve exterior, dispel irritability, and release stagnated heat	Regulate metabolic pathways, enhance cellular functions, inhibit pathogenic bacterial growth, and eliminate free radicals
*Massa Medicata Fermentata cum Galla*	*Chinese Gall*, Leaven, Tea	Gallic acid, tannin	Clear heat and resolve phlegm, promote fluid production and relieve thirst	Inhibit pro-inflammatory cytokines release, regulate metabolic pathways
*Massa Medicata Fermentata cum Pinellia*	Fresh Ginger, *Pinelliae Rhizoma Praeparatum Cum Alumine*, White Alum, *Massa medicata fermentata*	Enzyme, volatile oil composition	Relieves cough and resolves phlegm, promotes digestion and relieves stagnation	Inhibits mucus secretion and suppresses inflammatory factors
*Semen oryzae cum Monasco*	Rice, *Monascus purpureus*	Monacolin K, Monascus pigments, γ-aminobutyric acid	Promote digestion, harmonize the stomach, and activate blood circulation to resolve stasis	Regulate gastrointestinal hormone levels, improve intestinal microbiota structure, and inhibit HMG-CoA reductase activity

### Representative varieties of fermented Chinese medicine

#### *Arisaema cum Bile* (Dan Nan Xing)

*Arisaema cum Bile* is prepared from finely powdered *Arisaematis Rhizoma* (processed) and the bile of ox, sheep or pig, or prepared by fermentation of finely powdered *Arisaematis Rhizoma* with the bile of ox, sheep or pig. It has a bitter, slightly pungent taste and a cool nature, and belongs to the meridians of the lung, liver and spleen. As a traditional Chinese medicine, *Arisaema cum Bile* is often used to clear heat and resolve phlegm, calm wind and relieve convulsions.

##### Study on processing technology of *Arisaema cum Bile*

Market research has revealed that the fermentation processes of *Arisaema cum Bile* vary. The types of bile used as raw materials for fermentation are also diverse; they include not only the traditional raw material of ox bile, but also common pig bile, sheep bile, and pig bile paste, and the dosage of bile also differs by several times. The impact of different bile raw materials on its efficacy varies significantly. Moreover, the fermentation time ranges from 24 h to several months, and there are also differences in fermentation temperature and humidity. Since the fermentation of *Arisaema cum Bile* mostly relies on microorganisms in the natural environment, it is difficult to accurately control the fermentation process due to the influence of environment and seasonal changes [74, 77, 87]. Therefore, many scholars have studied the process parameters such as bile type, bile ratio, fermentation time, temperature and strain of *Arisaema cum Bile* to optimize the fermentation process.

A large number of studies have compared the anticonvulsant, antipyretic, and other effects of pig bile, ox bile, sheep bile, and the *Arisaema cum Bile* fermented by them. It was found that ox bile exhibits the optimal therapeutic effect, which is consistent with the traditional theory that “processing with ox bile eliminates dryness and intensity, rendering it clear and moistening.” [22, 23, 189]. The efficacy of bile and *Arisaematis Rhizoma* was compared by different ratios (3:1, 1:1, 1:3), and the results showed that the greater the proportion of bile, the better the antipyretic and expectorant and antitussive effects of *Arisaema cum Bile*, otherwise the effect was weakened [64]. However, while the higher proportion of bile can enhance therapeutic efficacy, its potential toxicity still requires further investigation. Researchers compared the bile acid components and anticonvulsant effects of *Arisaema cum Bile* fermented for 0, 3, 7, 15, and 30 days. It was found that with the extension of fermentation time, the content of free bile acids gradually increased while the content of conjugated bile acids decreased, indicating more complete fermentation. The anticonvulsant effect enhanced with the increase of fermentation days, and *Arisaema cum Bile* fermented for 15 days showed relatively stable properties and better efficacy. Meanwhile, studies on temperature and humidity conditions have revealed that temperature and humidity are crucial to the fermentation process. Both excessively low and high temperature and humidity can affect the fermentation process of *Arisaema cum Bile*, leading to incomplete conversion of conjugated bile acids [119]. In addition, based on traditional fermentation, some studies have isolated and screened dominant bacterial communities or strains, replacing traditional natural fermentation with strain fermentation. Finally, *Enterococcus* and *Enterococcus casseliflavus* were identified as the dominant composite strains for fermentation, which resulted in a higher content of bile acid components, the active ingredients, in *Arisaema cum Bile* compared to traditionally fermented products [108]. However, *Enterococcus* species pose risks of opportunistic pathogenicity and drug resistance, and *Enterococcus casseliflavus* lacks systematic toxicological data. Therefore, targeted safety evaluations are required to balance the synergistic effects of microbial fermentation with medication safety.

##### Study on chemical components of *Arisaema cum Bile*

*Arisaema cum Bile* has the effects of clearing heat and resolving phlegm, as well as calming wind and relieving convulsions. Its main chemical active components include various active ingredients such as polysaccharides, flavonoids, alkaloids and bile acids [12, 14, 104, 120]. The polysaccharide components in *Arisaema cum Bile* are basically consistent with those in *Arisaematis Rhizoma*, mainly composed of arabinose, xylose, mannose, glucose, and galactose [62]. However, the decomposition of glycosidic bonds during the processing, the polysaccharide content of *Arisaema cum Bile* is lower than *Arisaematis Rhizoma*, with a corresponding increase in monosaccharide content, and sugar derivatives are also produced. Similarly, the flavonoid components in *Arisaema cum Bile* were also from *Arisaematis Rhizoma*. After preparation, the flavonoid components would be decomposed. Compared with *Arisaematis Rhizoma*, the total flavonoids, schaftoside and isoschaftoside in *Arisaema cum Bile* were reduced to varying degrees [157, 158]. The alkaloid components in *Arisaema cum Bile* are consistent with those in *Arisaematis Rhizoma*. but these decrease significantly after water immersion [99], while there are no relevant reports on the impact of processing *Arisaematis Rhizoma* with bile on the content of alkaloids. During the fermentation process of *Arisaema cum Bile*, conjugated bile acids are converted into free bile acids. For example, glycohyocholic acid and taurohyocholic acid are converted into hyocholic acid; glycohyodeoxycholic acid and taurohyodeoxycholic acid are converted into hyodeoxycholic acid; while glycochenodeoxycholic acid and taurochenodeoxycholic acid are converted into chenodeoxycholic acid [76, 82, 85, 89]. In addition, the bile acid components in *Arisaema cum Bile* depend on the type of bile used. There are significant differences in the main components of *Arisaema cum Bile* processed with different types of bile. *Arisaema cum Bile* processed with ox bile mainly contains deoxycholic acid and cholic acid; that processed with pig bile mainly contains hyodeoxycholic acid, hyocholic acid, and chenodeoxycholic acid [84, 86], and that processed with sheep bile mainly contains deoxycholic acid [180].

##### Study on pharmacological effects of *Arisaema cum Bile*

After *Arisaematis Rhizoma* is processed into *Arisaema cum Bile* through bile fermentation, its dryness and toxicity are reduced, with its medicinal property transforming from warm to cool and its flavor changing from pungent to bitter [163, 174]. Studies on rat models of cold and heat syndromes have shown that *Arisaematis Rhizoma*, which is hot in nature, can increase the levels of serum indicators related to thyroid function. In contrast, *Arisaema cum Bile*, which is processed with bile and cool in nature, can reduce the levels of serum indicators related to metabolism in heat syndrome model rats. This confirms that the interaction between *Arisaematis Rhizoma* and bile brings about an essential change in medicinal property from hot to cold, thereby verifying the traditional theory of “reducing toxicity and modifying properties” in the fermentation processing of *Arisaematis Rhizoma* into *Arisaema cum Bile*, [109, 112, 113, 149].

In terms of antipyretic effect, *Arisaema cum Bile* prepared through different processes and from different bile sources effectively reduced the rectal temperature in high fever model rats, demonstrating definite antipyretic efficacy. This effect is associated with the modulation of Na⁺/K⁺-ATPase activity and the downregulation of inflammatory factors such as PGE₂. Moreover, fermentation processing can further enhance this antipyretic effect by improving microbial transformation efficiency. [62, 73, 83, 88, 189]. In terms of anticonvulsant effects, *Arisaematis Rhizoma* exhibits significant anticonvulsant activity but is highly toxic. After processing with bile, its toxicity is markedly reduced while the anticonvulsant activity is preserved and enhanced, prolonging the seizure latency and survival time in mice [70]. The fermented product demonstrates superior efficacy compared to the non-fermented product, and the effect of *Arisaema cum Bile* processed with ox bile was stronger than that with pig bile. This pharmacological effect is mediated through a synergistic neuro-immune dual pathway: on one hand, it regulates the homeostasis of neurotransmitters such as glutamate and GABA, mitigating hippocampal neuronal damage; on the other hand, it inhibits the activation of the TLR4/NF-κB pathway, reducing the release of pro-inflammatory factors such as IL-1β and TNF-α, thereby blocking the cascade amplification of neuroinflammation and convulsive injury [8, 13, 112, 113]. In the field of anti-inflammatory and analgesic effects, *Arisaema cum Bile* can elevate the pain threshold in mice. The bile acid components in its aqueous extract can scavenge DPPH and hydroxyl radicals, inhibit lipid peroxidation, thereby achieving a synergistic effect of antioxidant and analgesic actions [21]. The efficacy of *Arisaema cum Bile* is not due to isolated mechanisms, but rather the synergistic action of bile acid components. By blocking oxidative stress and reducing the release of inflammatory factors, it alleviates both hyperpyrexia and neuroinflammation, while mitigating inflammation-mediated neuronal damage and neurotransmitter imbalance. Ultimately, this achieves antipyretic, anticonvulsant, anti-inflammatory, and analgesic effects.

##### Study on quality evaluation of *Arisaema cum Bile*

At present, the preparation methods of *Arisaema cum Bile* are not uniform all over the country, resulting in the difficulty to control the quality of *Arisaema cum Bile*. In the previous editions of *Pharmacopoeia of the People’s Republic of China*, only character identification and physicochemical identification are included, while specific TLC identification items, content determination items for index components, inspection items, and limit requirements for extracts are lacking. This makes it impossible to accurately control the quality of *Arisaema cum Bile*, which needs further improvement. Therefore, many scholars have studied the principle of *Arisaema cum Bile* and developed an effective evaluation method to evaluate the quality of *Arisaema cum Bile*. Through chemical and pharmacological experiments, it was found that bile acid components were the active ingredients of *Arisaema cum Bile* [10]. Cholalic acid, hyodeoxycholic acid, chenodeoxycholic acid and deoxycholic acid are often used as index components, which can not only be used to evaluate the quality of *Arisaema cum Bile*, but also speculate the fermentation time and bile dosage through the content of these components. Generally, the longer the fermentation time or the higher the bile dosage, the higher the content of bile acid components [18]. In addition, components such as uracil, schaftoside and isoschaftoside are also used in the quality evaluation of *Arisaema cum Bile*. In terms of the exploration of comprehensive quality evaluation methods, some scholars have established a multi-component comprehensive quality evaluation model based on grey relational analysis model, which has enriched the quality evaluation method of *Arisaema cum Bile*.

#### *Massa Medicata Fermentata* (Liu Shen Qu)

The *Massa Medicata Fermentata* were first recorded as a drug name in the Tang Dynasty *Treatise on the Properties of Drugs*, which is a traditional compound fermented preparation made by fermenting together Polygonum hydropiper, wheat bran, flour, Phaseolus calcaratus, Armeniacae Semen Amarum, Artemisia annua and Xanthium sibiricum. It has a sweet and pungent taste and a warm nature, and belongs to the spleen and stomach meridians. The core effects are resolving food stagnation, regulating the digestive system and invigorating the spleen to harmonize the stomach.

##### Study on processing technology of *Massa Medicata Fermentata*

*Massa Medicata Fermentata* is a traditional Chinese medicine fermentation preparation. Traditional fermentation technology is widely used, but it has many limitations. In the traditional fermentation process, the degree of fermentation is often judged by experience, which is highly subjective, resulting in poor stability and repeatability of fermentation quality; in addition, in the traditional fermentation process, miscellaneous bacteria may be introduced, which affects the safety of *Massa Medicata Fermentata*.

At present, there are differences in the prescription and fermentation process of *Massa Medicata Fermentata* in different places, and there is no clear standard for fermentation temperature, humidity and time [31]. In terms of prescription selection, some scholars found through formula analysis that fresh *Massa Medicata Fermentata* was better than dry one for medicinal us (L. Wang, W. Gao, X. Pei, et al., 2017). Phaseolus calcaratus were used as fermented nitrogen source, and the optimal amount was 2.6 g flour with 100 g wheat bran [79]. In studies of fermentation cycle, scholars detected changes in the content of 5 enzymes during the fermentation of *Massa Medicata Fermentata* and compared the characteristic components of *Massa Medicata Fermentata* at different times by LC–MS. They concluded that the optimal fermentation cycle is 3 to 7 days [106, 166, 167, 169, 175]. In order to overcome the disadvantages of excessive miscellaneous bacteria and toxic metabolites in traditional fermented *Massa Medicata Fermentata*, some scholars proposed to use modern biotechnology to ferment *Massa Medicata Fermentata* with a single strain. Scholars conducted fermentation of *Massa Medicata Fermentata* using four strains isolated from self-made *Massa Medicata Fermentata* and two strains of Bacillus subtilis, respectively. They evaluated the quality of *Massa Medicata Fermentata* fermented by different pure strains based on enzyme activity and pharmacological activity, confirming that the quality of *Massa Medicata Fermentata* fermented by mucor genus of mold is the best [32]. Based on changes in enzyme activity and the content of active ingredients, many scholars have not only validated the single-strain fermentation methods using *Aspergillus flavus* [145] and *Aspergillus sydowii*, but also discovered co-fermentation methods with synergistic strains such as *Rhizopus oryzae* combined with *Saccharomycopsis fibuligera* [125, 136], and *Bacillus subtilis* combined with *Aspergillus sydowii* [94]. These pure-strain fermentation methods can not only ensure the quality of fermentation products, but also effectively avoid contamination by harmful bacterials and the production of toxic metabolites through pure culture fermentation, thereby guaranteeing the safety of *Massa Medicata Fermentata*.

##### Study on chemical components of *Massa Medicata Fermentata*

*Massa Medicata Fermentata* has the effects of promoting digestion and regulating the middle jiao, as well as strengthening the spleen and harmonizing the stomach. At present, a number of studies have found that the chemical composition of *Massa Medicata Fermentata* mainly comes from raw materials, such as quercetin, luteolin, arteannuin B (from *Artemisiae Annuae Herba*), polygoniin or isoorientin, and leucocyanidin (from *Polygoni Hydropiperis Herba*) [129, 138]. In addition to the components in the original formula, new substances are generated during fermentation. Comparative analysis of chemical composition changes before and after fermentation using NMR and HPLC revealed significant increases iin amino acids, alkenoic acids, ferulic acid, and protocatechuic acid during the fermentation process of *Massa Medicata Fermentata*. Conversely, components such as cynarin and scopoletin showed varying degrees of reduction. Notably, phenolic acids, amygdalin, and prunasin were undetectable post-fermentation. These findings suggest that these compounds do not constitute the pharmacologically active basis of *Massa Medicata Fermentata* [5, 30, 33, 93, 165]. In addition, *Massa Medicata Fermentata* also contains substances such as amylase, protease, and lipase. These are enzymes or metabolites produced by the metabolism of microorganisms during the fermentation process. These substances can decompose the starch and protein in *Massa Medicata Fermentata* into small molecules that are easily absorbed by the human body, thereby endowing *Massa Medicata Fermentata* with the effects of promoting digestion and invigorating the spleen. The raw materials of *Massa Medicata Fermentata* do not originally contain enzyme substances like amylase and protease; it is precisely through fermentation that these enzymes are generated.

##### Study on pharmacological effects of *Massa Medicata Fermentata*

*Massa Medicata Fermentata* are digestive fermented medicines, clinically commonly used to treat food stagnation and abdominal distension. Modern pharmacological studies have mostly focused on exploring the mechanisms of its digestion-promoting effect, such as enhancing gastrointestinal motility, promoting the secretion of gastrointestinal hormones, and restoring the imbalance of intestinal flora. In terms of promoting digestion, studies have confirmed that *Massa Medicata Fermentata* can enhance the contraction of ileal smooth muscles and promote small intestinal peristalsis. Moreover, its effect is stronger than unfermented *Massa Medicata Fermentata*. A study on the serum components of mice with dyspepsia models found that *Massa Medicata Fermentata* can increase the contents of gastrin and cholinesterase [171, 172]. In terms of intestinal flora, studies have shown that *Massa Medicata Fermentata* can increase the levels of *Bifidobacterium* and *Bacteroides* while reducing the number of *Enterobacter* and *Enterococcus*. As an intestinal microecology regulator, it promotes the growth of beneficial anaerobic bacteria for the body like *Bifidobacterium*, maintains intestinal balance, and regulates microbial composition [129, 138]. Furthermore, it can regulate the intestinal flora of mice with spleen deficiency, improve intestinal tissue damage, and exert a protective effect against organ damage caused by flora imbalance [146] (L. Wang, W. Gao, X. Xu, et al., 2017). Studies have shown that *Massa Medicata Fermentata* promotes cell proliferation in RAW264.7 and IEC-6 cells, enhances immune organ index in animals, and elevates levels of IL-6 and TNF-α, thereby improving the body's immune function. In conclusion, the *Massa Medicata Fermentata* can promote gastrointestinal motility, regulate intestinal flora and exhibit anti-inflammatory effects, but its mechanism of action needs to be further elucidated to guide rational clinical drug use.

#### *Sojae Semen Praeparatum* (Dan Dou Chi)

*Sojae Semen Praeparatum* is a fermented product made from the mature seeds of legume Glycine max (L.) Merr. It is often processed with *Morus Folium* and Artemisiae Annuae Herba as auxiliary materials. Historically, *Sojae Semen Praeparatum* was first recorded under the name *Chi*, an ancient Chinese term originally referring to fermented soybeans, in *Mingyi Bielu*, a classic and pivotal text of traditional Chinese medicine. It has a bitter and pungent taste and a cool nature, and belongs to the lung and stomach meridians. It is good at relieving exterior symptoms, resolving restlessness and diffusing stagnated heat.

##### Study on processing technology of *Sojae Semen Praeparatum*

The fermentation of *Sojae Semen Praeparatum* is a complex process, which is affected by many factors such as Chinese herbal raw materials and fermentation technology. The production of *Sojae Semen Praeparatum* typically uses either black or yellow soybeans as raw materials. Modern research indicates that black soybeans are richer in nutrients such as starch, protein, and carbohydrates, which are more conducive to microbial growth and fermentation compared to yellow soybeans. Many studies using different legumes as raw materials for making *Sojae Semen Praeparatum* have found that those produced from black soybeans demonstrate higher nutritional value [76, 81, 82, 85, 89]. Key indicators like soy isoflavones show that fermentation of black soybeans enables maximum conversion of glycosides into aglycones, achieving a “thorough extraction” level. In terms of medicinal efficacy, fermented black beans also exhibit superior advantages over their yellow soybean counterparts [49]. The above conclusions are primarily based on specific cultivars. Variations in germplasm composition, cultivation environments, and harvesting periods among different black bean varieties may influence substrate nutrient profiles and fermentation processes. Future studies should incorporate both cultivar-specific and cultivation-related factors for multidimensional validation. *Sojae Semen Praeparatum* is primarily produced through a two-stage fermentation process: the pre-fermentation allows microbial communities and enzymes to penetrate into the beans, increasing acidity and darkening the color.;the post-fermentation enzymatic hydrolysis of macromolecules forms the flavor profile, and appropriate temperature elevation can shorten the production cycle [141, 179, 182].

##### Study on chemical components of *Sojae Semen Praeparatum*

*Sojae Semen Praeparatum* is skilled at relieving exterior symptoms, resolving restlessness and diffusing stagnated heat. The mainly components, including protein, soy isoflavones, fat, polysaccharides, play an important role in its efficacy [72]. Before fermentation, active components in soybeans such as isoflavones and soyasaponin primarily exist in glycosylated forms, which are poorly absorbed by the human body. After fermentation, these compounds undergo decomposition. The levels of isoflavone glycoside and genistin decrease, while those of isoflavone aglycone and genistein increase. Notably, the total isoflavone content remains unchanged, yet bioavailability significantly improves [69, 72]. In the later fermentation process, the content of isoflavone aglycone increased, while other isoflavones decreased [103]. Furthermore, the fermentation of soybeans produces new substances such as GABA, melanoidins, and biogenic amines. Among these components, GABA, an important inhibitory neurotransmitter in the central nervous system, is formed during the “re-steaming and aging” process of the natural fermentation of *Sojae Semen Praeparatum*. Specifically, it is produced via the decarboxylation of glutamic acid, catalyzed by glutamic acid decarboxylase [11, 19]. The content of GABA is closely related to factors such as pH value, temperature, the activity of glutamic acid decarboxylase, and the levels of neutral and alkaline proteases [12, 14]. Furthermore, during the fermentation of *Sojae Semen Praeparatum*, fibrinolytic enzyme is also produced [96]. This enzyme not only exhibits high fibrinolytic activity but also possesses excellent anticoagulant and thrombolytic effects, and can be directly absorbed by the digestive tract. Multiple studies have also found that after fermentation of soybeans, the content of phenolic compounds, antioxidant components, increases significantly [9, 16, 17, 39]. When researching the polysaccharide components of *Sojae Semen Praeparatum* at different fermentation stages, it was observed that the mass fraction of polysaccharides increases with the extension of time in the early stage of fermentation, gradually decreases as fermentation time progresses during the fermentation process, and then shows a gradual upward trend [74, 77, 87].

##### Study on pharmacological effects of *Sojae Semen Praeparatum*

*Sojae Semen Praeparatum* have a variety of pharmacological effects, including hypoglycemic, hypolipidemia, prevention of osteoporosis, anticancer, promotion of bone cell proliferation, antibacterial, thrombolytic and other pharmacological effects. In terms of lipid-lowering effects, animal models of hyperlipidemia were established to study the lipid-lowering function of soy isoflavones—the active component of *Sojae Semen Praeparatum*. The results showed that soy isoflavones can regulate blood lipids by increasing indicators such as hepatic high-density lipoprotein cholesterol and lipoprotein lipase in the liver. Meanwhile, clinical studies have demonstrated that *Sojae Semen Praeparatum* can significantly improve the lipid levels of patients with lung adenocarcinoma [55]. In terms of hypoglycemic effects, the n-butanol extract of *Sojae Semen Praeparatum* can reduce blood glucose levels in streptozotocin-induced diabetic rats challenged with glucose and improve glucose tolerance. Fermentation products of *Sojae Semen Praeparatum*, such as soy isoflavones and β-galactosidase, have been confirmed to exert hypoglycemic effects [116, 188]. In the context of osteoporosis treatment, cell-based experiments demonstrated that isoflavone components in *Sojae Semen Praeparatum* are effective in promoting osteoblast proliferation, differentiation, and bone matrix formation [110, 153, 154]. Furthermore, the ethyl acetate fraction of *Sojae Semen Praeparatum* exhibits the strongest antibacterial activity, and some of the compounds isolated from this fraction have been confirmed to possess antibacterial properties [41]. The isoflavone components and polysaccharides of *Sojae Semen Praeparatum* exhibit antioxidant activities—such as scavenging DPPH free radicals—through distinct mechanisms. These components can effectively inhibit the damage to the human body caused by reactive oxygen species free radicals, thereby contributing to human antioxidant defense and alleviating aging (Xu, Huang, et al., 2020). In summary, *Sojae Semen Praeparatum* are rich in components such as isoflavones and amino acids, and exhibit significant therapeutic effects. In-depth research into its mechanisms of action and expansion of its clinical applications can provide insights and evidence for the development of functional foods and the prevention and treatment of related diseases.

#### *Massa Medicata Fermentata cum Galla* (Bai Yao Jian)

*Massa Medicata Fermentata cum Galla* is a traditional fermented Chinese medicinal decoction piece. It is a block-like substance covered with a “white frost” on the surface, fermented from raw materials such as *Galla chinensis*, tea leaves, and distiller's yeast under natural conditions. It has a sour and astringent taste and is cold in nature, affecting the lung, stomach, and large intestine meridians, with the effects of moistening the lungs to resolve phlegm, relieving fever, and promoting fluid production [59].

##### Study on processing technology of *Massa Medicata Fermentata cum Galla*

Across different regions, the processing technologies of *Massa Medicata Fermentata cum Galla* vary in terms of distiller's yeast, auxiliary materials, and formula ratios. Specifically, white liquor distiller’s yeast is used in Beijing, while rice wine distiller's yeast is adopted in Jiangxi Province. Regarding the selection of auxiliary materials, the “Wen School”—a traditional processing faction—in Wuhan uses black tea and smoked plum decoction, whereas the “Jing School”—another traditional processing faction—employs auxiliary materials such as licorice root, platycodon grandiflorus, and green tea. Green tea, as one of the raw materials for *Massa Medicata Fermentata cum Galla*, has its processing and addition methods significantly affect the content of gallic acid. Comparative studies on the effects of tea leaves, tea juice, and tea residue on fermentation reveal that when the residue from boiled tea leaves and tea juice are combined as fermentation substrates, the tannin conversion rate becomes higher and the fermentation effect improves significantly [107]. Wine yeast serves as a critical factor influencing fermentation efficiency. Since different strains contain varying microbial species and quantities, their ability to enzymatically break down tannins and produce gallic acid differs significantly. Research indicates that using Angelic Wine Yeast during fermentation may result in white mold formation and “vineyard blooming” phenomena, while also demonstrating enhanced gallic acid production capabilities [7]. Additional studies have shown that fermenting with strains such as *Rhizopus* (e.g., *Rhizopus oryzae*) alone, or via co-fermentation with *Bacillus megaterium strain*, *Bacillus cereus*, *Hyphopichia burtonii*, and *Kluyveromyces marxianus*, can both increase the active component content of gallic acid in *Massa Medicata Fermentata cum Galla* and optimize fermentation efficiency [43].

##### Study on chemical components of *Massa Medicata Fermentata cum Galla*

The core material basis for *Massa Medicata Fermentata cum Galla* to exert its astringent, hemostatic, and lung-moistening effects is gallic acid, which is converted from hydrolyzable tannins in *Galla chinensis* through microbial fermentation processing [44]. It has the activity of killing cone worms, anti-inflammatory, antibacterial, anti-tumor and antiviral, so the efficacy can be preliminarily judged by measuring the content of gallic acid in *Massa Medicata Fermentata cum Galla* [43, 127, 128, 132, 137]. The main raw material of *Massa Medicata Fermentata cum Galla* is gallnut, which is rich in tannin components. Therefore, research on the chemical components of *Massa Medicata Fermentata cum Galla* has focused primarily on the tannic component, gallic acid. In the gastrointestinal tract, tannins can bind to proteins on the mucosal surface to form macromolecular precipitates, which irritate the gastrointestinal tract and cause adverse reactions. However, the fermentation and processing of Chinese gallnut can reduce such irritation.

##### Study on pharmacological effects of *Massa Medicata Fermentata cum Galla*

*Galla chinensis* has a sour and astringent taste and a cold nature in TCM theory. It can alleviate symptoms in rat models of “heat syndrome” induced by levothyroxine sodium. After *Galla chinensis* is fermented into *Massa Medicata Fermentata cum Galla*, its taste and nature undergo changes. Studies have shown that both *Galla chinensis* and *Massa Medicata Fermentata cum Galla* can significantly improve energy metabolism disorders in rats and inhibit the excitability of the central nervous system and endocrine system. Notably, the effect of *Galla chinensis* is superior to that of *Massa Medicata Fermentata cum Galla*, which indicates that the fermented *Massa Medicata Fermentata cum Galla* has a milder cold nature and altered medicinal properties [130, 134]. The pharmacological effects of *Massa Medicata Fermentata cum Galla* before and after fermentation were compared, and it was found that the anti-inflammatory, analgesic, cough suppressant and sputum transformation effects were enhanced after fermentation. The anti-inflammatory mechanism may be the inhibition of TNF-α, IL-6 and IL-1β production [11, 19, 131, 133]. In terms of intestinal flora, *Massa Medicata Fermentata cum Galla* can improve intestinal flora dysbiosis by regulating the relative abundance and diversity of intestinal flora, protect the intestinal mucosal barrier function, and thereby exert a role in alleviating intestinal inflammation [131, 133].

#### *Massa Medicata Fermentata cum Pinellia* (Ban Xia Qu)

The fermentation method for preparing *Massa Medicata Fermentata cum Pinellia* originated as early as the Song Dynasty. It is a fermented product made from *Pinellia Rhizoma Praeparatum Cum Alumine* combined with fresh ginger juice, alum, flour, and Medicated Leaven, and is one of the common processed products among traditional Chinese medicine fermented preparation. It has a pungent taste and a warm nature, and belongs to the spleen, stomach and lung meridians. It combines the effects of *Pinellia ternata* (Thunb.) Breit.of drying dampness to resolve phlegm and lowering adverse qi to stop vomiting, with the effects of fermented agents of promoting digestion and harmonizing the stomach.

##### Study on chemical components of *Massa Medicata Fermentata cum Pinellia*

After fermentation, *Massa Medicata Fermentata cum Pinellia* produces digestive enzymes such as amylase and protease, which are directly related to its medicinal effect of promoting digestion and resolving food stagnation. Meanwhile, fermentation also affects the formation of volatile oil components, mainly including fatty acids, sesquiterpenes and monoterpenes; these substances are regarded as the important material basis for the medicinal effects of *Massa Medicata Fermentata cum Pinellia* in eliminating phlegm and relieving asthma [25]. In addition, the glycyrrhizic acid components undergo dynamic transformation during the fermentation process: with the progress of fermentation, the content of glycyrrhizic acid decreases gradually, while the content of glycyrrhetinic acid shows a changing trend of first increasing and then decreasing. This further reflects the profound impact of microbial fermentation on the formation of medicinal components [98].

##### Study on pharmacological effects of *Massa Medicata Fermentata cum Pinellia*

A comparison of the effects of Pinelliae Rhizoma, and *Massa Medicata Fermentata cum Pinellia* reveals that after Pinelliae Rhizoma is fermented into *Massa Medicata Fermentata cum Pinellia*, while retaining its original functions of drying dampness to resolve phlegm, lowering adverse qi to stop vomiting, and relieving stuffiness to disperse masses, it not only enhances the effect of drying dampness to resolve phlegm but also gains new effects of invigorating the spleen, warming the stomach, promoting digestion, and relieving food stagnation. In experimental studies, evaluations based on mouse gastrointestinal—related indicators such as gastrin, cholinesterase, and nitric oxide showed that the promoting effect of fermented *Massa Medicata Fermentata cum Pinellia* on gastrointestinal function was significantly stronger than that before fermentation, with a marked improvement in digestive function. This confirms that it can be used to treat the syndrome of spleen deficiency and food stagnation [115, 117]. The fermentation process of Chinese medicine is not only capable of moderating the property of medicines, enhancing or altering their efficacy, but also of great significance in reducing their toxic and side effects. Pinelliae Rhizoma itself is pungent in taste, warm in nature, and toxic. Ancient people believed that when it is co—fermented with auxiliary materials to make *Massa Medicata Fermentata cum Pinellia*, its toxicity is moderated. Modern studies further indicate that the irritation of Pinelliae Rhizoma mainly comes from calcium oxalate raphides and the proteins bound to them, among which lectin proteins can intensify the irritating effect of the raphides [67]. After fermentation, the content of calcium oxalate raphides in *Massa Medicata Fermentata cum Pinellia* is relatively reduced, and its irritation to the conjunctiva of rabbits' eyes is significantly weakened. This proves that the fermentation process for making *Massa Medicata Fermentata cum Pinellia* effectively moderates the irritation of Pinelliae Rhizoma.

#### *Semen oryzae cum Monasco* (Hong Qu)

*Semen oryzae cum Monasco* is made by fermenting rice inoculated with *Monascus*. Throughout history, it has been widely used in food processing fields such as liquor brewing [185]and sufu (fermented bean curd) production [105], serving as a coloring and flavoring agent. It holds high economic value and broad market prospects, and is renowned as the “natural statin”. *Semen oryzae cum Monasco* has a sweet taste and a warm nature, and belongs to the spleen, large intestine and liver meridians. It has the effects of promoting digestion and harmonizing the stomach, as well as activating blood circulation to dissipate blood stasis [148].

##### Study on processing technology of *Semen oryzae cum Monasco*

The fermentation of *Semen oryzae cum Monasco* is a comprehensive process driven by the combined action of multiple factors. Its product quality is influenced by process parameters such as microbial strains, fermentation medium, and pH value and duration during fermentation. Modern optimization of *Semen oryzae cum Monasco*’s fermentation processing technology also mostly focuses on these three aspects, aiming to increase the yield of specific products auch as monacolin K, Monascus pigments, or γ-aminobutyric acid and reduce the production of toxic components citrinin.

Since the ChP 2015 edition, *Semen oryzae cum Monasco* has been defined as a product prepared by artificially culturing *Monascus purpureus Went strain* (CGMCC No. 0272)—a fungus belonging to the family Trichocomaceae—inoculated onto rice (hulled kernels). Strains are the core of fermentation. In order to obtain *Monascus strains* with high yields of Monascus pigments and low yields of citrinin, studies have isolated and purified the *Monascus strain M7-5* through expanded strain screening [159]. Additionally, mutagenesis breeding of *Monascus purpureus strain M2* can also yield optimal mutant strains with high Monascus pigment production: the pigment-producing capacity of these mutant strains reaches 2.3 times that of the original strain, and they maintain stable high yields across generations.

The fermentation medium serves as the foundation for microbial cell growth and product synthesis; a suitable medium formulation can enhance the yield of target products and ensure more stable product quality. Optimization of the medium typically focuses on aspects such as the type, dosage, and ratio of carbon and nitrogen sources, the type and dosage of inorganic salts, as well as certain exogenous additives [164]. Different types of additives yield distinct effects, and selecting the optimal additives can maximize product output. Studies have shown that L-arginine can increase the yield of monacolin K and alter the degree of indentation and folding in mycelia [78, 171, 172], sodium acetate can also significantly enhance the yield of lovastatin [4, 163, 174]. Therefore, during the fermentation of *Semen oryzae cum Monasco*, researchers used *Monascus purpureus* as the fermentation strain and rice as the fermentation substrate. They added 0.60% L-arginine and 0.15% sodium acetate to the solid fermentation substrate at different fermentation stages, supplemented with appropriate nutrients. After optimizing the fermentation conditions, the yield of acid-form monacolin K increased by 153.85% [15]. In addition, studies have found that adding 7% glycerol and 2% sodium nitrate during the fermentation process yields the optimal process conditions for achieving high Monascus pigment production and low citrinin production [159].

The optimization of fermentation process parameters is based on temperature, humidity, pH and fermentation time. It was found that the variable temperature fermentation technology could significantly increase the yield of Monacolin K. The common variable temperature fermentation scheme was 30℃ for the first 3d and 26℃ for the second 3d [176]. The pH plays an important role in the production of metabolites in *Semen oryzae cum Monasco* fermentation. When the pH value of the fermentation broth is higher, the yield of Monacolin K, the effective component of red yeast rice, increases first and then decreases [100]. The control and optimization of fermentation time can indirectly affect the production of metabolites. At the 24th day of solid-state culture, the yield of acid Monacolin K reached the maximum value. With the extension of fermentation time, the nutrient substrate in the solid-state culture medium was exhausted, and the yield of Monacolin K decreased [118].

##### Study on chemical components of *Semen oryzae cum Monasco*

*Semen oryzae cum Monasco* has the effects of promoting digestion and harmonizing the stomach, as well as activating blood circulation to dissipate blood stasis. Its components mainly include metabolites of Monascus purpureus, such as monascus pigments, Monacolin K, citrinin, and stigmasterol [66].

**Fig. 3:**
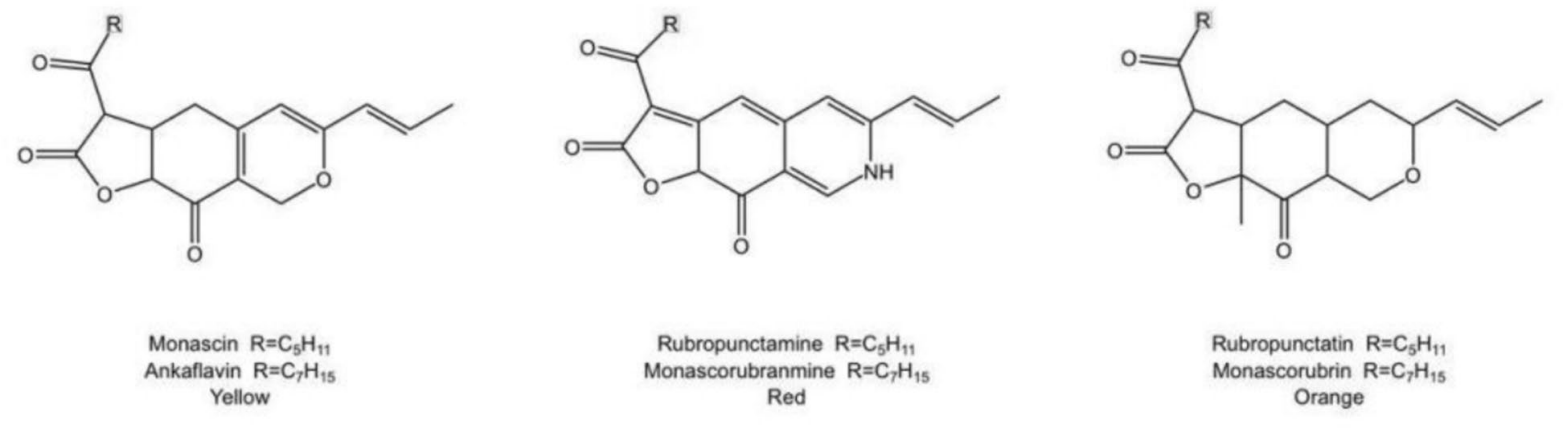
Structure of main pigment types in *Semen oryzae cum Monasco*

Monascus pigments are a class of polyketide compounds and high-quality natural food colorants produced by Monascus fermentation. According to reports, the synthesis of Monascus pigments occurs during the metabolic process of Monascus [2]. Multiple highly active intermediates are present in their biosynthetic pathway, which can undergo multi-step disproportionation reactions. As a result, a diverse range of Monascus pigment products are formed, with the main pigment structures shown in Fig. 3 [54]. Compared with synthetic colorants, Monascus pigments is stable, heat resistance, safe, non-toxic, and non-teratogenic, which are the ideal natural food additive. In addition to these advantages, Monascus pigments also possess bioactivities including antibacterial, lipid-lowering, and anti-cancer effects [1, 186].

Monacolin K is a statin component. Owing to its structural similarity to 3-hydroxy-3-methylglutaryl-coenzyme A reductase in the human cholesterol synthesis pathway, it can competitively inhibit this enzyme to block cholesterol synthesis and reduce cholesterol activity, thereby directly exerting a lipid-lowering effect. It is the main lipid-lowering active component in *Semen oryzae cum Monasco* [162, 187]. Since the open-loop acidic form of monacolin K in functionally fermented natural *Semen oryzae cum Monasco* has high bioavailability and directly determines lipid-lowering efficacy, the content of acid-form Monocolin K has also become a critical indicator for evaluating the lipid-lowering effect of *Semen oryzae cum Monasco* [161].

**Fig. 4:**
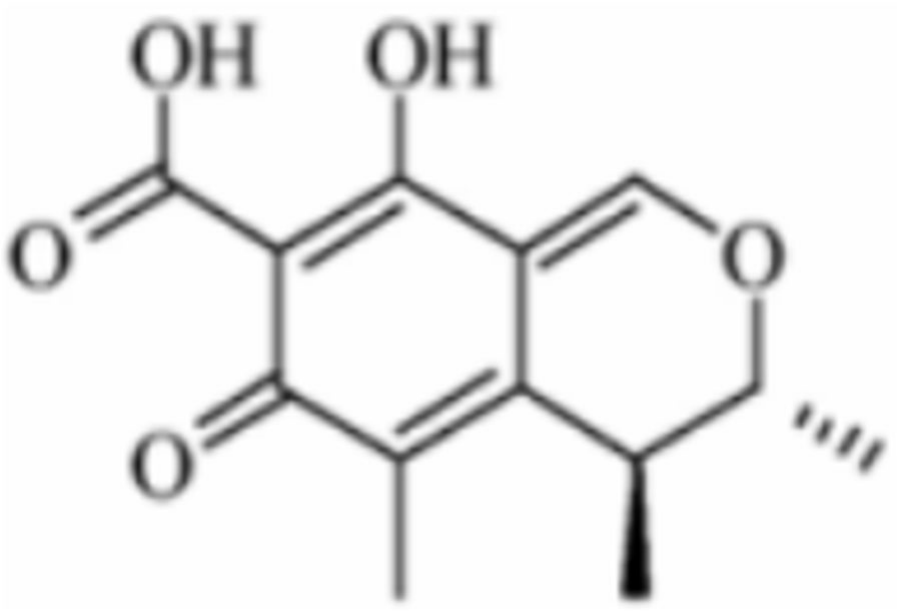
Structure of citrinin

Citrinin derives its name from being first discovered in the culture broth of Penicillium citrinum, and its structure is shown in Fig. 4. Citrinin exhibits significant nephrotoxicity, which can cause renal tubular dilation, degeneration, and necrosis of epithelial cells in animals. Among other effects, it also possesses reproductive toxicity and embryotoxicity [61]. In 1995, French scholars Blanc et al. found that some Monascus strains produced harmful mycotoxin citrinin in the metabolic process, and the safety of *Semen oryzae cum Monasco* products has been widely concerned [56, 65, 126, 135, 139]. To standardize the quality of *Semen oryzae cum Monasco*, China has established a limit for citrinin contamination in *Semen oryzae cum Monasco* products in accordance with GB 1886.181—2016 National Food Safety Standard for Food Additive *Semen oryzae cum Monasco*. This standard specifies that the citrinin content per unit color value of *Semen oryzae cum Monasco* shall be ≤ 0.04 mg/kg. Notably, the safety risks of *Semen oryzae cum Monasco* products do not stem solely from citrinin. In 2024, *Semen oryzae cum Monasco* products from Kobayashi Pharmaceutical were detected to contain puberuic acid, which also drew attention to the safety issues of *Semen oryzae cum Monasco*.

##### Study on pharmacological effects of *Semen oryzae cum Monasco*

In traditional Chinese medicine, *Semen oryzae cum Monasco* is credited with the effects of promoting digestion and blood circulation, as well as invigorating the spleen and nourishing the stomach. Modern pharmacological studies have shown that it exhibits multiple biological activities, including lowering blood glucose, reducing blood lipids, anti-atherosclerosis, anti-osteoporosis [143], anti-inflammation [144, 147], and anti-cancer effects. Compared with ordinary rice, *Semen oryzae cum Monasco* has a stronger effect on diabetes treatment. *Semen oryzae cum Monasco* has a more significant effect on improving blood glucose level, weight and adipose tissue index of diabetic mice, which proves that rice fermentation into *Semen oryzae cum Monasco* has a better effect on diabetes treatment. In addition, *Semen oryzae cum Monasco* can improve the gastrointestinal function of mice with spleen deficiency and food retention by regulating gastrointestinal hormone levels, improving gastrointestinal tissue morphology, and restoring intestinal flora structure [91]. Monacolin K, the main component of *Semen oryzae cum Monasco*, can reduce cholesterol levels. Multiple studies have shown that *Semen oryzae cum Monasco* can significantly improves lipid metabolism disorders in rats fed a high-fat diet: it lowers serum total cholesterol, triglyceride, and low-density lipoprotein cholesterol levels, and alleviates hepatic lipid accumulation. Its mechanism of action not only includes inhibiting the activity of HMG-CoA reductase (similar to the mechanism of monacolin K alone) but also exhibits a unique lipid-lowering pathway through the synergistic effects of multiple active components [157, 158]. Additionally, *Semen oryzae cum Monasco* can effectively inhibit alcohol-induced activation of NF-κB in hepatocytes and hepatocyte apoptosis [168], as well as reduce atherosclerotic lesions in mice [26], providing a new research direction for the prevention and treatment of metabolic diseases [40].

## Preparation technology of Chinese medicine sprouting

### History of preparation technology of Chinese medicine sprouting

**Fig. 5:**
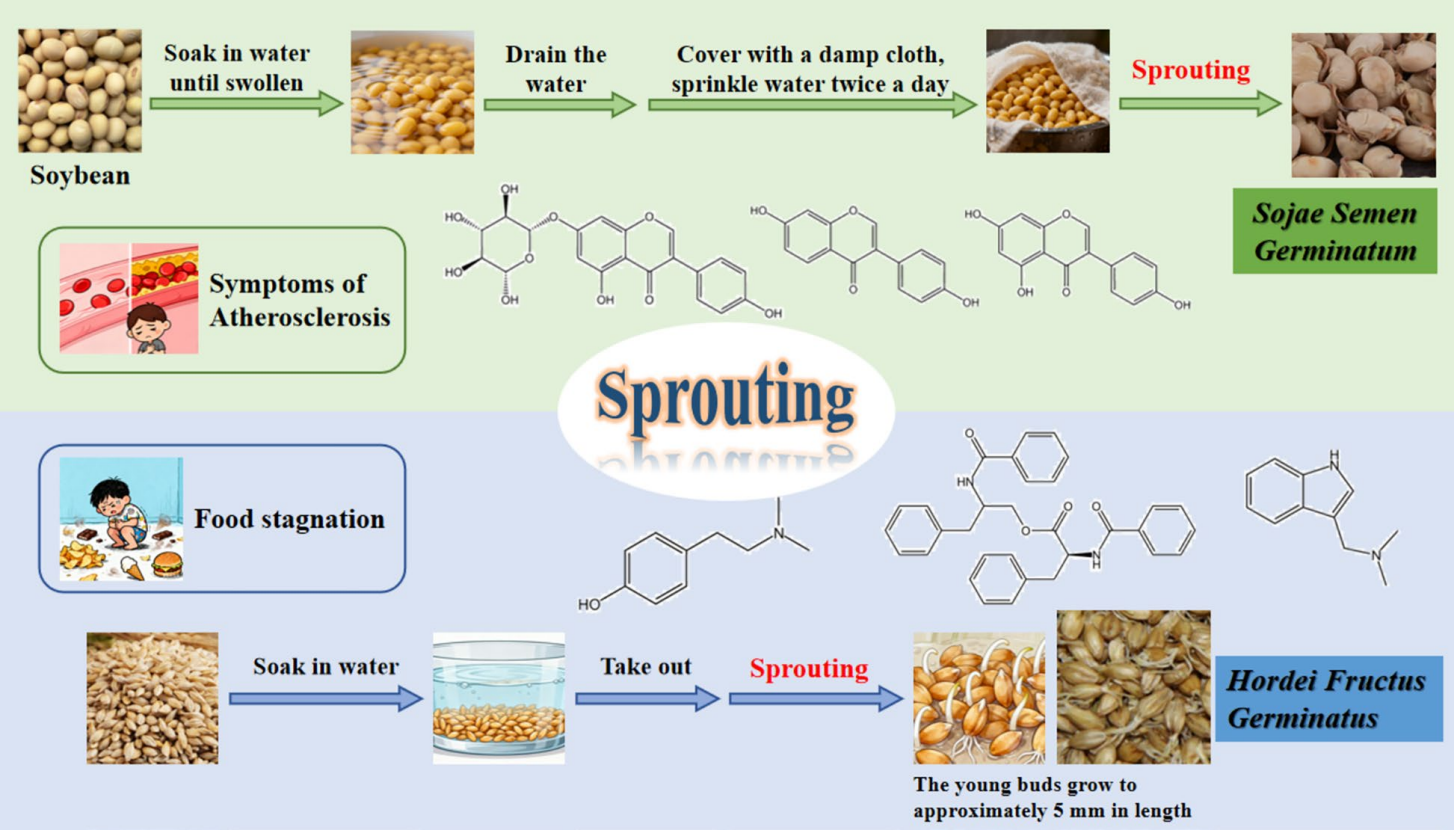
Representative varieties of traditional sprouting of Chinese medicine

The sprouting processing method of TCM has a long history and was known as *Nie Fa* in ancient times, which involves germinating selected fruits or seeds under suitable temperature and humidity conditions to alter their original properties, generate new therapeutic effects, and expand the variety of processed medicinal materials [52]. This method is primarily applied to fruits or seeds, most commonly serving as staple food crops in China, such as *Sojae Semen Germinatum* from germinated soybeans and *Hordei Fructus Germinatus* from germinated barley (Fig. 5). Before the Tang Dynasty, traditional Chinese medicine materials prepared via sprouting were mostly used in their raw form. *Jinkui Yaolue* records the use of raw *Sojae Semen Germinatum* ground into powder, while *Xinxiu Bencao* provides a detailed account of the sprouting process for making *Sojae Semen Germinatum* using soybeans as the raw material. Subsequently, traditional Chinese medicine began to emphasize the impact of processing methods (raw or cooked) on the efficacy of sprouted materials, leading to the development of various processing techniques suitable for clinical diseases, including grinding, stir-frying, decoction, and vinegar treatment and other processing approaches [9, 16, 17, 115, 117]. Compared with other processing methods such as stir-frying, steaming, and fermentation, the sprouting method holds unique technological and pharmacological significance. Sprouting is a mild endogenous enzyme-driven biotransformation process that requires neither intense heating nor exogenous auxiliary materials. This approach not only prevents the loss of heat-sensitive active components but also activates endogenous hydrolytic enzyme systems. Sprouting can convert macromolecular nutrients such as starch and proteins into easily absorbable functional secondary metabolites, including oligosaccharides and amino acids, thereby enhancing the utilization rate and efficacy of medicinal materials. This represents an advantage that cannot be achieved through simple heating or physical processing [51]. This paper will provide a comprehensive and in-depth review of *Hordei Fructus Germinatus* and *Sojae Semen Germinatum* representative sprouting Chinese medicine, in order to provide useful reference for related research and clinical application.

### Representative varieties of sprouted Chinese medicine

#### *Hordei Fructus Germinatus* (Mai Ya)

*Hordei Fructus Germinatus* is a processed preparation of the mature fruits of Hordeum vulgare L., an annual plant in the Poaceae family, after sprouting and drying. It has a sweet taste and neutral nature, affecting the spleen and stomach meridians. It effectively promotes qi circulation to aid digestion, strengthens the spleen to stimulate appetite, and alleviates lactation and abdominal distension.

##### Study on processing technology of *Hordei Fructus Germinatus*

The sprouting processing involves the following steps: first, soak barley in water; then maintain appropriate temperature and humidity; when the young buds grow to approximately 5 mm in length, dry the barley in the sun or at a low temperature to obtain raw *Hordei Fructus Germinatus*. During this process, the activity of enzymes such as amylase and protease in the seeds increases significantly, which promotes the decomposition of components like starch into maltose—theese forms the material basis for *Hordei Fructus Germinatus*'s function of aiding digestion [92]. The total alkaloid content, the content of barley betaine and the activity of amylase were taken as indicators. The sprouting conditions were optimized by orthogonal experiment to improve the amylase activity by 30% compared with the traditional method [38]. Now, on the basis of traditional technology, equipment such as constant temperature and humidity incubator and fermentation tank are used to accurately control the temperature, humidity and time of sprouting, which not only improves the uniformity of bud length, but also reduces microbial contamination [173].

##### Study on chemical components of *Hordei Fructus Germinatus*

After malt is processed into *Hordei Fructus Germinatus*, the effects of promoting qi circulation to aid digestion and invigorating the spleen to improve appetite are enhanced, and the chemical composition has a significant change [3, 75, 80, 122]. Among the enzymes, α-amylase and β-amylase are the main types: the former hydrolyzes starch into dextrin, while the latter further decomposes dextrin into maltose, thereby promoting the body's absorption of nutrients [37, 45]. As important components of *Hordei Fructus Germinatus*, polyphenols include ferulic acid, gallic acid, protocatechuic acid, etc., and most of them exist in the form of conjugated phenolic acids. The flavonoid components mainly include quercetin, catechin, kaempferol, among which the content of these compounds increases to varying degrees after barley sprouting [122]. *Hordei Fructus Germinatus* contains 17 kinds of amino acids such as glycine, γ -aminobutyric acid and glutamic acid. The content of free amino acids increases obviously during sprouting [124, 144, 147].

##### Study on pharmacological effects of *Hordei Fructus Germinatus*

Research on the medicinal effects of *Hordei Fructus Germinatus* has mainly focused on promoting digestion to resolve food stagnation, suppressing lactation, and promoting lactation. *Hordei Fructus Germinatus* is a traditional Chinese medicine with bidirectional regulatory effects on milk secretion. Studies suggest that this bidirectional regulatory action is associated with alkaloid content [130, 134]. In high-dose, *Hordei Fructus Germinatus* can reduce the number of PRL-positive cells in the pituitary gland, downregulate PRL cell mRNA expression, and activate the DRD2-protein kinase A signaling pathway, significantly inhibiting PRL levels and suppressing abnormal milk secretion [34, 121]. While low-dose *Hordei Fructus Germinatus* can increase the number and volume of mammary lobules in lactation-deficient female mice, promote ductal dilation and secretion of abundant milk, elevate PRL levels, and improve lactation capacity [150]. Clinically, for patients with insufficient milk secretion, a small amount of *Hordei Fructus Germinatus* is administered to promote the flow of qi in the mammary glands, thereby increasing milk production. Conversely, for those with patent milk ducts and excessive milk secretion requiring galactoestasis, a larger dose of *Hordei Fructus Germinatus* is typically prescribed [46]. Nutritional components such as hordenine and hemicellulose contained in *Hordei Fructus Germinatus* can significantly alleviate the symptoms of ulcerative colitis by improving the intestinal microenvironment, repairing the intestinal mucosal barrier, and increasing the level of SCFAs [47, 123]. The rich phenolic compounds in *Hordei Fructus Germinatus* can reduce the level of transaminase such as alanine transaminase and aspartate transaminase in serum of mice with liver injury, enhance the activity of antioxidant enzymes such as superoxide dismutase, reduce hepatocyte apoptosis and damage, and show the liver-protective potential of malt [102].

##### Study on quality evaluation of *Hordei Fructus Germinatus*

The quality evaluation of *Hordei Fructus Germinatus* mostly takes germination length as an indicator. The Chinese Pharmacopoeia (2025 Edition) has clear regulations on the germination rate and length of *Hordei Fructus Germinatus*: the germination rate shall not be less than 85%, and the bud length shall be approximately 5 mm. It also sets a limit on aflatoxin, ensuring the safety of clinical medication. During the sprouting process of *Hordei Fructus Germinatus*, the activities of α-amylase and β-amylase increase. The level of enzyme activity is directly related to the digestive function of *Hordei Fructus Germinatus*, while the types and contents of volatile components are closely associated with *Hordei Fructus Germinatus*’s flavor, medicinal effects, and other properties [8, 13, 35]. Therefore, some studies believe that enzymatic activity and volatile components can be used to determine the sprouting time and evaluate the quality of *Hordei Fructus Germinatus*.

#### *Sojae Semen Germinatum* (Da Dou Huang Juan)

*Sojae Semen Germinatum* is obtained by sprouting and drying the mature seeds of Glycine max (L.) Merr., also known as Dadou Huangjuan in Chinese. It tastes sweet and has a neutral nature, acting on the spleen, stomach, and lung in TCM theory. Its functions include relieving exterior symptoms and dispelling summer-heat, as well as clearing heat and promoting diuresis.

##### Study on processing technology of *Sojae Semen Germinatum*

**Fig. 6:**
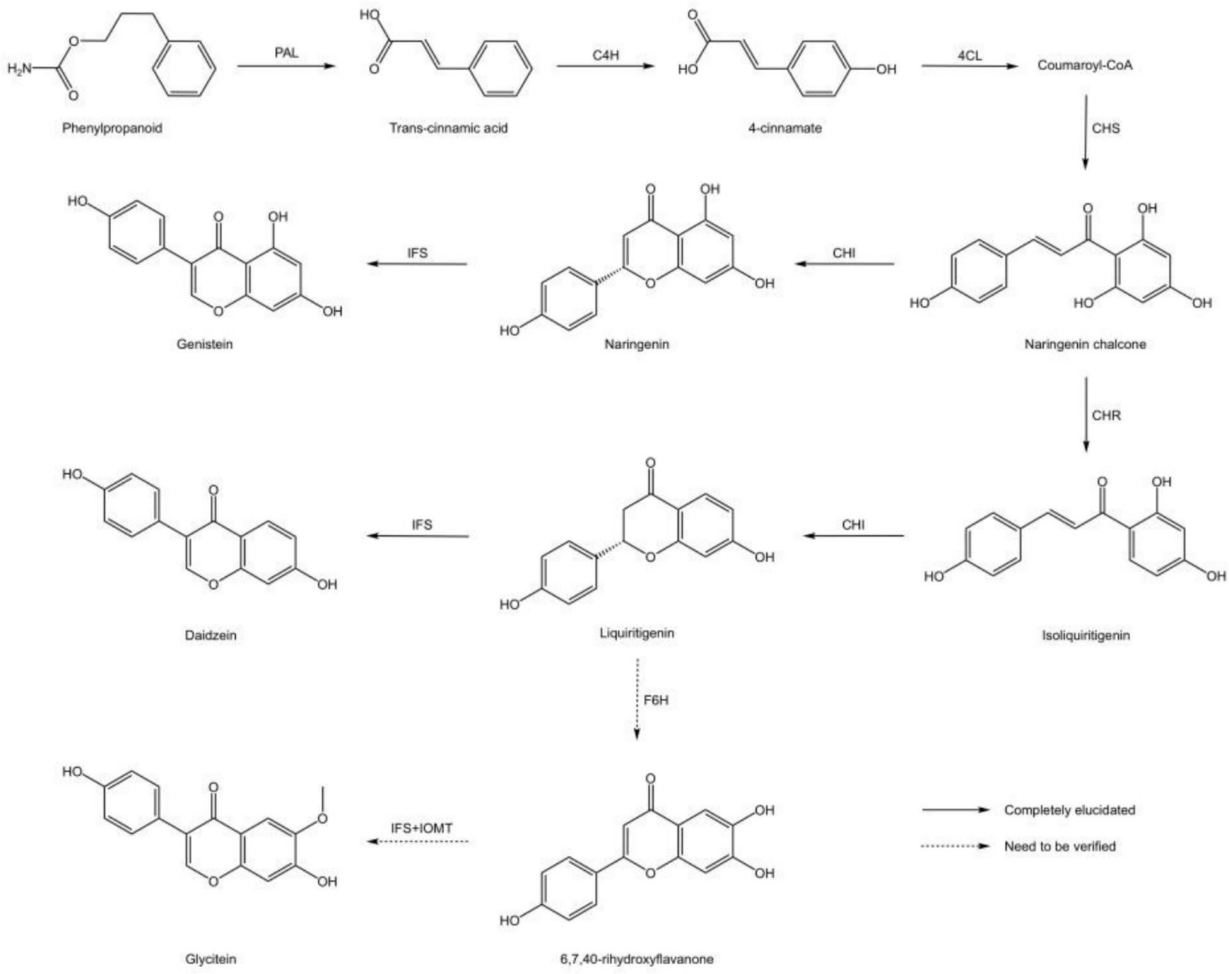
Biosynthesis pathways of soy isoflavones

*Sojae Semen Germinatum* has the effects of releasing the exterior to dispel summer-heat and clearing heat to promote diuresis. The main chemical components including proteins, phospholipids, saponins, isoflavones, amino acids, vitamins, and various minerals and trace elements[9, 16, 17]. As processed soybean products, *Sojae Semen Germinatum* share similar chemical compositions with soybeans, yet differ in specific types and concentrations. Current research on the bioactive components of *Sojae Semen Germinatum* primarily focuses on soybean isoflavones, including soybean glycosides, anthraquinone, and genistein. The synthesis pathways are illustrated in Fig. 6 [95]. During the sprouting process of soybeans into *Sojae Semen Germinatum*, nutritional components such as proteins, carbohydrates, fats, and carbohydrates are consumed. Additionally, reactions such as the conversion of soy isoflavone glycosides to aglycones and the degradation of proteins into polypeptides occur. These changes lead to certain alterations in the types and contents of chemical components in *Sojae Semen Germinatum*. Experiments have confirmed that soybean sprouting can increase the contents of components such as soy isoflavones, soy saponins, free amino acids, soy polypeptides, soluble proteins, reducing sugars, vitamin C, γ-aminobutyric acid, and free trace elements. At the same time, it reduces the contents of anti-nutritional factors such as phytic acid and trypsin inhibitors, as well as the contents of total sugars and lipoxygenase [48].

##### Study on quality evaluation of *Sojae Semen Germinatum*

Modern research has shown that soy isoflavones in *Sojae Semen Germinatum* are recognized as chemical components with strong biological activity, among which genistin is present in relatively high content; meanwhile, sprouting significantly increases the content of leucine. Based on this, the Chinese Pharmacopoeia (2025 Edition) has established quality control regulations for *Sojae Semen Germinatum*: thin-layer chromatography is used for the qualitative identification of leucine and genistin, and under the “content determination” section, it clearly requires that the total content of daidzin and genistin, measured by high-performance liquid chromatography, shall not be less than 0.08%. During the sprouting process, reactions such as the conversion of isoflavone glycosides to aglycones and the degradation of proteins into polypeptides occur, and the contents of components such as soy saponins, vitamin C, and γ-aminobutyric acid also increase (Yeming & C, 2015). Therefore, there are certain limitations in achieving quality control of *Sojae Semen Germinatum* solely by controlling the contents of leucine, daidzin, and genistin [9, 16, 17]. It is necessary to apply modern scientific and technological methods to conduct in-depth research on the quality evaluation of *Sojae Semen Germinatum* so as to reasonably and effectively control its quality and efficacy, and lay a foundation for its clinical application.

## Preparation technology of directional fermentation technology

In the modernization of TCM, the application scope of fermentation technology has been significantly expanded, extending from traditional fermented TCM to the fermentation of non-traditional medicinal materials such as *Polygonatum* and *Lactobacillus Picp-2*, *Pueraria lobata* and *Aspergillus niger*, and *Panax notoginseng* and *Lactobacillus plantarum*. Directional fermentation, which precisely controls microbial metabolism through artificial conditions, features low energy consumption, short processing time, and mild conditions. It not only enhances the efficacy of TCM, reduces toxicity, and improves pharmacokinetic properties but also overcomes the limitations of traditional fermentation, which has a narrow application range. This represents a significant innovation in fermentation technology for TCM modernization [58, 63, 126, 135, 139, 166, 167]. At present, there are three kinds of directional fermentation: single-strain fermentation, multi-strain synergistic fermentation and bidirectional fermentation. The following is a review of the modern research progress of three fermentation modes in the field of Chinese medicine fermentation and processing, and a systematic review of their technical breakthroughs and mechanism innovations in the transformation of Chinese medicine components, regulation of medicinal properties, and quality improvement, so as to provide reference for the theoretical deepening and industrial application of Chinese medicine fermentation and processing (Table 3).

### Single-strain fermentation

Single-strain fermentation refers to a technology that uses only one screened and optimized microbial strain for fermentation during the TCM fermentation process. With its characteristics of high controllability and easy standardization, this technology has been widely applied in the field of TCM fermentation. Currently, the commonly used microorganisms in single-strain fermentation technology include *lactic acid bacteria*, *yeast*, and *Bacillus subtilis*.

*Lactic acid bacteria* can ferment carbohydrates to produce large amounts of organic acids such as lactic acid and acetic acid. During the fermentation of *Crataegi Fructus*, the organic acids metabolized by lactic acid bacteria can regulate the pH value of the fermentation system. This not only inhibits the growth of harmful microorganisms but also promotes the release and transformation of active components such as total flavonoids in hawthorn, significantly enhancing its antioxidant capacity [24, 90]. In the *Puerariae Lobatae Radix* fermentation system, *lactic acid bacteria* can decompose macromolecular substances such as cellulose and starch in *Puerariae Lobatae Radix*, thereby enhancing the bioavailability of *Puerariae Lobatae Radix* alkaloids. In the experiments of T2DM and hyperlipidemia model rats, it was found that fermentation could regulate energy metabolism, lipid metabolism and amino acid metabolism, thereby enhancing its hypoglycemic and hypolipidemic effects [27, 84, 86]. During this process, the metabolic products of lactic acid bacteria promote component transformation, while the bacterial cells maintain fermentation equilibrium. The synergistic interaction between these two processes enhances the pharmacological effects [73, 83, 88, 156]. *Yeast* is a single-celled fungus that can survive in both aerobic and anaerobic environments, with strong metabolic capabilities. In the fermentation system of *Lycii Fructus*, *yeast* can utilize the carbohydrate substances in *Lycii Fructus* for fermentation, producing ethanol, esters, and other substances, altering the original aroma of *Lycii Fructus* and giving it a unique flavor [178]. In addition, various enzymes secreted by *yeast* can break down the polysaccharides, proteins, and other components in *Lycii Fructus* into smaller molecules that are more easily utilized by the human body, thereby enhancing the nutritional value and antioxidant capacity of *Lycii Fructus*. Studies have shown that *Lycii Fructus* fermented by *yeast* showed a clearance rate of DPPH free radicals and hydroxyl radicals as high as 92.3% and 95.84%, respectively, while the inhibition rate of α-glucosidase was significantly increased by 30.23%. Its antioxidant and hypoglycemic effects were significantly improved compared to the original product [60, 101]. *Bacillus subtilis* can produce a variety of biologically active enzymes and metabolites. The spores it contains quickly consume oxygen in the animal intestinal tract after entering it, maintaining an anaerobic environment, which promotes the growth of beneficial anaerobic bacteria and inhibits the growth of harmful aerobic bacteria. Meanwhile, it can also increase the levels of immunoglobulins and antibodies, enhance cellular immunity and humoral immune functions, and improve the group immunity. In the fermentation system of *Polygonati Rhizoma* and *Bacillus subtilis*, the fermentation product significantly enhances the thymus and spleen indices in mice, prolongs the swimming exhaustion time, reduce serum MDA levels, and increases hepatic glycogen levels. Additionally, it eliminates the irritant and tongue-numbing effects of the raw product, while improving its anti-fatigue and antioxidant properties, as well as enhancing its sweetness and aroma [36, 76, 82, 85, 89].

Although single-strain fermentation technology offers the advantage of strong controllability, its actual fermentation efficacy is highly dependent on strain stability. If genetic mutations occur during strain passage, it may lead to decreased enzyme activity and altered metabolites, thereby affecting the quality uniformity of fermentation products. Future efforts should focus on enhancing the stability of single strains through strain improvement to promote the standardized application of single-strain fermentation technology in traditional Chinese medicine processing.

### Multi-strain synergistic fermentation

Multi-strain synergistic fermentation is a technology that combines two or more types of microorganisms for the fermentation of traditional Chinese medicine. This method aligns with traditional Chinese medicine fermentation models, offering advantages such as rapid fermentation rates and efficient nutrient utilization [74, 77, 87].

In the study of honeysuckle seed fermentation, *Lactobacillus acidophilus* and *Bacillus subtilis* were were co-fermented in a 1:1 ratio. The lactic acid produced by *Lactobacillus acidophilus* reduces the system's pH, inhibits contamination by heterotrophic bacteria, and simultaneously promotes the secretion of enzymes by *Bacillus subtilis*. These enzymes specifically hydrolyze the glycosidic bonds of flavonoids in honeysuckle seeds, converting bound chlorogenic acid and luteolin into free forms. Both compounds were present in higher concentrations compared to fermentation using either *Lactobacillus acidophilus* alone or *Bacillus subtilis* alone, confirming the existence of a synergistic effect. Pharmacological experiments demonstrated that the mixed fermentation broth significantly enhanced the protective effects on alcoholic liver injury model mice: compared to the model group, serum levels of ALT, AST, and MDA were reduced, SOD activity was increased, and intestinal microbiota balance was regulated. These findings confirm that co-fermentation enhances hepatoprotective and microbiota-regulating activities [183, 184]. Despite the significant advantages of multi-strain co-fermentation, risks such as complex metabolites remain. Future efforts should focus on elucidating the metabolic interaction networks among strains and integrating detection methods like fingerprinting to achieve precise quality control of products.

### Bidirectional fermentation

Bidirectional fermentation involves inoculating medicinal fungi onto a TCM material-based culture medium. Under suitable conditions, the medicinal fungi utilize the nutrients in the TCM materials for growth and reproduction; meanwhile, their metabolites act on the TCM materials to transform the chemical components of the TCMs. In turn, the TCM materials provide nutrients for the growth of the medicinal fungi and may influence the metabolic pathways of the medicinal fungi, prompting the fungi to produce new bioactive components. This fermentation process, characterized by mutual interaction and mutual influence, enables bidirectional transformation between the medicinal fungi and the TCM materials, hence the name “bidirectional fermentation” [111].

The most prominent feature of bidirectional fermentation is that it fully leverages the respective advantages of medicinal fungi and TCM materials, achieving “fungi-TCM” synergistic enhancement of efficacy. In the solid-state fermentation system of *Astragali Radix* using the medicinal *fungus Ganoderma*, the enzymes secreted by *Ganoderma* during its growth convert astragaloside IV, the active monomer in astragalus residue, into isoastragaloside IV. This transformed component can more effectively promote the phagocytic function of macrophages and enhance the body's immune system. After the bidirectional fermentation of *Polygoni Multiflori Radix* and *Ganoderma*, the contents of active substances such as polysaccharides, triterpenes, and total flavonoids in *Polygoni Multiflori Radix* are significantly increased through the metabolic action of *Ganoderma*. In vitro antioxidant experiments have shown that its scavenging capacity against DPPH free radicals and superoxide anion free radicals is significantly enhanced, exhibiting high antioxidant activity [152]. In the fermentation system of *Isatidis Radix* and *Auricularia heimuer*, the metabolic activity of *Auricularia heimuer* induces structural changes in the polysaccharides of *Isatidis Radix*; meanwhile, the content of inosine produced by *Auricularia heimuer* itself increases. These changes enhance the anti-tumor and anti-inflammatory activities of the fermented product [76, 82, 85, 89].

TCM constitutes a complex chemical system, and the introduction of microbial strains further increases its complexity. Therefore, in the construction of TCM fermentation systems, it is essential to first select one or more appropriate microbial strains based on TCM theory as the fermentation strains. Fermentation should not be conducted arbitrarily, and full consideration must be given to the compatibility between the fermentation substrate and the microbial strains. With the innovation of fermentation technology, traditional quality requirements based on characteristics such as color and shape can no longer be used to evaluate the quality of fermented TCM. Establishing a comprehensive quality control standard based on traditional characteristic features, enzyme activity, hygiene, and limits of harmful chemical substances is a crucial guarantee for the safe and effective clinical application of fermented TCM.

## Conclusions

This article systematically reviews the effects of TCM fermentation technology and sprouting technology on TCM, focusing on the optimization of processing techniques, the transformation laws of TCM components, the mechanisms of efficacy changes, and quality evaluation methods. Meanwhile, it summarizes emerging technologies such as single-strain fermentation, multi-strain synergistic fermentation, and bidirectional fermentation, expands the application scope of fermentation processing, and injects new vitality into the modernization of traditional TCM processing technology. This not only clearly sorts out the internal connection between biological processing technology, TCM component changes, and efficacy regulation, but also provides a reference for in-depth exploration of the mechanism of action of biological processing technology, optimization of biological processing technology to give full play to the clinical value of TCM, and promotion of the modernization and industrialization of TCM processing (Table 3).

**Table 3:** Preparation technology of directional fermentation technology

Fermentation type	Traditional Chinese Medicine	Strain	Core changes before and after fermentation	Core strength of fermentation	Reference documentation
Single-strain Fermentation	*Bupleuri* Radix- *Scutellariae* Radix	*Aspergillus niger*	Saikosaponin A/D, baicalin, baicalein, wogonin increase, boosting anti-inflammatory and antipyretic effects	Increase the content of active components; nhance efficacy	[29]
	*Polygonati* Rhizoma	*Lactobacillus plantarum NX-1*	Increased pancreatic lipase inhibition rate, elevated polysaccharides, saponins and specific metabolites, significantly inhibiting lipid accumulation in high-fat zebrafish	Enhance efficacy	[6]
	*Glycyrrhizae* Radix Et Rhizoma	*Lactobacillus plantarum ZKLp100*	Inhibits the TLR4/NF-κB pathway and reconstitutes intestinal flora. Reconstituting the intestinal flora, the effect of alleviating colitis is enhanced	Enhance efficacy	[42]
	*Puerariae Lobatae* Radix	*Aspergillus niger*	Xylanase and carboxymethyl cellulose enzyme from fermentation degrade cell wall fibers, promoting intracellular puerarin release	Efficiently prepare puerarin and enhance the utilization value of *Puerariae Lobatae* Radix residue	[166, 167]
	*Puerariae Lobatae* Radix	*Eurotium cristatum*	The contents of isoflavone components puerarin and daidzein increase, while the content of daidzin decreases	Enhance the content of bioactive substances and antioxidant activity	[28]
	*Notoginseng* Radix Et Rhizoma	*Lactobacillus plantarum*	Optimization of the fermentation process, with enhanced immunomodulatory effects	Enhance efficacy	[58, 63]
	*Polygonati* Rhizoma	*Lactobacillus Picp-2*	Increased DPPH・ and other free radical scavenging rates, total reducing power, and α-glucosidase inhibition	Enhance efficacy	[126, 135, 139]
	*Puerariae Lobatae* Radix	*Eurotium cristatum*	The content of active substances and antioxidant activity were increased	Enhance the accumulation of active substances and antioxidant capacity	[28]
	*Magnoliae Officinalis* Cortex	*Aspergillus niger*	Enhanced tyrosinase inhibition, antioxidant activity and other multi-activities	Enhance efficacy	[68]
	*Gegen Qinlian* Decoction	*Yeast*	Act on the p38MAPK pathway and exhibits a better hypoglycemic effect in rats with type 2 diabetes mellitus	Enhance efficacy	[56, 65]
	*Puerariae Lobatae* Radix	*probiotic*	Enhanced antioxidant enzyme activity in liver/related tissues and reduced lipid peroxidation products better alleviate alcoholic liver injury	Enhance efficacy	[142]
	*Gegen Qinlian* Decoction	*Yeast*	Reduces gluconeogenesis, increases glucose utilization, decreases non-medicinal components, and increases active components	Increase the content of active ingredients; enhance efficacy	[27]
	*Puerariae Lobatae* Radix	*Escherichia coli*	Prolong the lifespan of Caenorhabditis elegans, promote egg-laying, increase heat-resistant time, inhibit the expression of daf-2, up-regulate daf-16, and promote the expression of hsp-16.2 and sod-3	Enhance efficacy	[177]
	*Gegen Qinlian* Decoction	*Yeast*	Protects the lung and colon tissues of influenza-infected mice, reduces viral expression in lung tissue, and inhibits the expression of TLR-7 to exert an anti-inflammatory effect	Enhance efficacy	[151]
	*Phellinis*	*Yeast*	Exhibit different inhibitory effects on HepG2, Hela, and HCT-116 cells after fermentation	Enhance efficacy	[140]
	*Astragali* Radix	*Yeast*	Outperforms the unfermented counterpart in regulating pulmonary microcirculation, alleviating inflammation, and improving lung function in PM2.5-induced lung injury rats	Enhance efficacy	[73, 83, 88]
	*Astragali* Radix	*Yeast*	The fermented product of Astragalus membranaceus can weaken the near-infrared fluorescence signal in mice with acute lung injury and reduce the expression of TNF-α, IL-1, IL-6 and TLR4	Enhance efficacy	[75, 80]
	*Astragali* Radix	*Yeast*	The fermented product of *Astragali* Radix on the TLR4-Src-NFκB-ICAM-1 pathway in rats with lung injury, and its regulatory effect on this pathway is superior to that of unfermented *Astragali* Radix	Enhance efficacy	[74, 77, 87]
	*Gegen Qinlian* Decoction	*Yeast*	Increases antioxidant enzyme activity, reduces lipid peroxides and ROS production, enhances free radical scavenging capacity in T2DM rats, with better oxidative stress improvement and pancreatic β-cell protection than the unfermented group	Enhance efficacy	[57]
Multi-strain Synergistic Fermentation	*Astragali* Radix	*Bifidobacterium breve and Bifidobacterium infantis*	The content of astragaloside IV decreases, while the content of astragaloside II increases	Alters the proportion of various bioactive components	[71]
	*Lonicerae Japonicae Flos-Cassiae Semen*	*Lactobacillus acidophilus and Bacillus subtilis*	Total polysaccharides, flavonoids, saponins and other bioactive components are significantly increased; fermented products show better alcoholic liver disease protection and intestinal flora regulation than unfermented extracts	Increase the content of active ingredients; enhance efficacy	[183, 184]
	*Puerariae Lobatae* Radix	*Bifidobacterium breve*	Fermentation significantly downregulates intestinal MCP-1, IL-6, TNF-α gene expression; AMPK phosphorylation activation reduces adipocyte size	Enhance efficacy	[20]
	*Puerariae Lobatae* Radix	*Lactobacillus plantarum、Lactobacillus paracasei*	Significant relieve the alcohol-induced discomfort and regulation of alcohol metabolism-related indicators	Enhance efficacy	[9, 16, 17]
	*Ginseng* Radix Et Rhizoma	*Bacillus subtilis、Lactobacillus rhamnosus、Lactobacillus casei*	The *Ginseng* Radix Et Rhizoma extract fermented by compound strains increases the proliferation rate of mouse hair follicle cells	Enhance efficacy	[126, 135, 139]
Bidirectional Fermentation	Highland Barley	*Ganoderma mycelium*	Optimization fermentation technology; Increase the content of triterpene under optimal conditions	Enhance efficacy	[97]
	*Astragali* Radix	*Poria*	Better uric acid regulation and renal function protection in hyperuricemic mice than the unfermented raw materials	Enhancing efficacy	[53]

## Limitations and future prospects

As an important field integrating traditional processing technology and modern biotechnology. Biological processing of TCM is expected to achieve breakthrough progress in basic research, technological innovation, and application expansion in the future.

At the fundamental research level, fermentation processing is essential to reveal the relationships among microorganisms, TCM, and active components through multi-omics technologies such as genomics, transcriptomics, and metabolomics, thereby elucidating the metabolic pathways of key functional microbial communities and the transformation patterns of pharmacophores. Sprouting processing requires the application of molecular biology and metabolomics techniques to rigorously quantify the effects of endogenous enzyme, temperature, humidity, and light on the bioactive components. The core breakthrough in future basic research lies in achieving a transition from “phenomenological description” to “mechanistic elucidation,” providing quantifiable scientific evidence for technological optimization.

In terms of technological innovation, fermentation processing should establish clear standards for strain selection, preservation conditions, and application protocols. The development of intelligent control systems for fermentation parameters such as temperature, humidity, and pH value is essential to address issues like inconsistent inoculation quantities and poor controllability during fermentation. For sprouting processing, modern agricultural technologies should be leveraged to establish standardized cultivation and sprouting systems. Key steps, including seed pretreatment and environmental regulation during sprouting, should be optimized to achieve uniform quality and large-scale production of processed raw materials.

In terms of standardization and regulatory framework development, a comprehensive quality control system must be established. For quality evaluation, integrated assessment models encompassing fingerprinting, multi-component quantification, and bioactivity evaluation should be implemented, with clearly defined quality control indicators and limit standards for different processing methods. At the regulatory level, it is essential to improve the filing system for processing techniques, establish safety protocols for microbial strain usage, and enhance product traceability systems. These measures will strengthen risk prevention against microbial contamination and harmful substance generation during biological processing.

Moving forward, the advancement of biological processing of TCM requires deeper integration of basic research and practical applications, emphasizing interdisciplinary innovation with technologies like artificial intelligence. Simultaneously, potential safety risks must be critically evaluated. By achieving a balance between innovation and regulation, high-quality development can be realized, providing core technological support for the modernization and internationalization of TCM.

## Data Availability

No datasets were generated or analysed during the current study.

## Abbreviations

CDCA: Chenodeoxycholic acid
ChP: Chinese Pharmacopoeia
GABA: Gama-aminobutyric acid
HCA: Hyocholic acid
HDCA: Hyodeoxycholic acid
HMG-CoA: 3-Hydroxy-3-methylglutaryl-coenzyme A
HPLC: High performance liquid chromatography
IL-6: Interleukin 6
IL-1β: Interleukin 1β
LC-MS: Liquid Chromatography-Mass Spectrometry
MCP-1: Monocyte Chemoattractant Protein-1
NF-κB: Nuclear transcription factor-κB
NMR: Nuclear Magnetic Resonance Spectroscopy
PM2.5: Particulate Matter with Aerodynamic Diameter ≤ 2.5 μm
ROS: Reactive oxygen species
TCM: Traditional Chinese Medicine
TLC: Thin Layer Chromatography
TLR4: Toll-like receptor 4
TLR7: Toll-like receptor 7
T2DM: Type 2 Diabetes Mellitus
TNF-α: Tumor Necrosis Factor-α
